# *Noncoding RNA 886* alleviates tumor cellular immunological rejection in host C57BL/C mice

**DOI:** 10.1002/cam4.3148

**Published:** 2020-05-31

**Authors:** Hui Ma, Miao Wang, Ying Zhou, Jia‐jie Yang, Li‐Yong Wang, Rong‐hui Yang, Min‐jie Wen, Lu Kong

**Affiliations:** ^1^ Department of Biochemistry and Molecular Biology Capital Medical University Beijing China; ^2^ Department of Pathology Beijing Friendship Hospital The Second Clinical Medical College of Capital Medical University Beijing China; ^3^ Core Facilities for Molecular Biology Capital Medical University Beijing PR China

**Keywords:** cancer biology, epigenetics, immunology, metastasis, prostate cancer

## Abstract

Non‐coding RNA 886 (nc886/VTRNA2‐1) is a Pol III transcript and an atypical imprinted gene. Its exact function as a negative regulator of protein kinase R establishes its connection with innate immunity. Studies have shown that nc886 silencing is closely associated with prostate cancer progression. Previous work has constructed a cell model of stable nc886 overexpression (“mimic” or “nc886^+^”) in PC‐3M‐1E8 cell lines (1E8), which are highly bone‐metastatic human prostate cancer cells with low expression of nc886, and cells expressing the mimic were validated to have lower invasive and metastatic abilities than cells expressing the scramble transcript in vitro and in vivo. In this study, we directly injected mimic or scramble cells into the left ventricle of C57BL/C mice, an immunocompetent animal model, to elucidate the immune mechanisms of tumor‐host interactions. Interestingly, we found that tumor cells induced the inflammation of many important organs due to xenogeneic antigen rejection; this inflammation was ultimately repaired by tissue fibrosis after 28 days, except for in the spleen. The reason is that mimic cells, as heterogeneous antigens, are mostly directly recognized by macrophages or T cells in blood, and few mimic cells enter the spleen compared with scramble cells. The induction of splenic macrophage polarization to M2 macrophages by scramble cells is a critical factor in maintaining chronic splenic inflammation. In addition, we recognize that nc886 broadly decreases the expression of some human leukocyte antigen molecules and antigen transporters. This evidence reveals the interesting role of nc886 in regulating tumor cell antigens.

## Graphical abstract

Nc886 broadly decreases the expression of some human leukocyte antigen molecules and antigen transporters, establishing the interesting role in regulating tumor cell antigens. Nc886 alleviates the inflammatory response of acute rejection caused by human‐derived prostate cancer cellular xenografts in immunocompetent mouse models. Nc886 reduces the polarization of macrophages through TGF‐β1 inhibition.

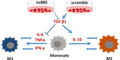

## 1 INTRODUCTION

*nc886,* as a “metastable epiallele”, is a maternally methylated gene in humans.^1^ However, this gene is lost in mice or rats.^2^ Its aberrant methylation is closely associated with various cancers.^3^ In human prostate cancer, *nc886* is considered as a tumor suppressor.^4^ Earlier studies reported that *nc886* regulated PKR and 2',5'‐oligonucleotide synthetase 1.^5^ King et al found that *nc886* was a marker of T cell activation and that its transcriptional activation depended on IFN‐γ and IL‐12 produced by activated macrophages under conditions of viral infection.^6^ Accidently, we noticed that *nc886* alleviated the inflammatory response of acute rejection caused by human‐derived prostate cancer cellular xenografts in immunocompetent mouse models. Hence, this study focuses on the immunomodulatory role of *nc886* in prostate cancer cell lines. A few studies have reported the mechanism of the tumor cell‐mediated immune response.

Cancer immunoediting research in immunodeficient hosts is obviously insufficient.^7^ However, even tumor cells from allogeneic or xenogeneic backgrounds can be rejected by immunocompetent host animals due to major histocompatibility complex (MHC) gene mismatches.^8^ MHC antigens and human leukocyte antigens (HLAs) are the most important known targets of the rejection response.^9^ More than 200 genes of the HLA complex are clustered on chromosome 6 and divided into three types: type I (HLA‐A, ‐B, and ‐C), type II (HLA‐DP, ‐DQ, and ‐DR), and type III (complement components, tumor necrosis factor, heat shock protein 70, and MHC class I‐related chain A, B).^10^ Antigen presentation in antigen presenting cells(APCs) often requires the help of some antigen processing genes that are localized between the HLA class I and II genes, such as the *TAP1*, *TAP2*, *LMP2*, *LMP7*, *DMA*, and *DMB* genes. In addition, damage‐associated molecular patterns (DAMPs) produced from dying cells have been recently identified as triggers of xenograft rejection.^11^ To date, the best‐known DAMPs include high‐mobility group box‐1 (HMGB1)**, **S100A8** **and S100A9** **(calgranulin A and B), and serum amyloid A (SAA).^12^

Inflammation induced by rejection is a complex immune process involving adaptive and innate immunity.^13^ Undoubtedly, macrophages are the main cells that mediate acute rejection within 1‐2 days, and the M2 phenotype was recently shown to be the predominant phenotype in xenogeneic rejection.^14^ The polarization of macrophages is tremendously plastic, and their phenotype status generally depends on local environmental molecules.^15^ M1‐type macrophages are frequently classified as activated macrophages and M2 as alternatively activated macrophages.^16^ In general, M1 macrophages are involved in killing bacteria, fungi, and viruses via the secretion of IL‐1, IL‐6, TNF‐α, and IL‐23, and M2 macrophages repair injured tissue by increasing the expression of arginase‐1 (Arg‐1), mannose receptor (CD206), IL‐10, and transforming growth factor β (TGF‐β).^15^ Additionally, M2 macrophages in the tumor microenvironment, considered tumor‐associated macrophages (TAMs), have an immunosuppressive role that promotes tumor metastasis.^17^ Allograft rejection within 1‐2 weeks primarily is a result of CD8^+^ cytotoxic T cells activated by type I alloantigens, which assist CD4^+^ helper T cells in recognizing type II alloantigens. The CD8‐MHC I system can mediate early cellular rejection though direct T cell allorecognition. The CD4‐MHC II system recognizes processed alloantigens on the surface of APCs and may be important in maintaining chromic rejection.^10^ Growing evidence has shown that xenogeneic antigens can also initiate T‐cell‐mediated direct or indirect rejection.^18^

HLA molecules are highly polymorphic, and HLA‐A, HLA‐B, and HLA‐DRB1 are recognized to be closely related to allogeneic rejection^19^.^20^ Interestingly, our RNA‐sequencing data analysis found that *nc886* downregulated the expression of some mRNAs, including *HLA‐A*, *HLA‐DRB1*, and*TAP1*, in human prostate cancer cell lines. However, in humans, MHC I/II loss or downregulation on tumor cells often contributes to immune escape, metastatic progression and a poor prognosis in numerous malignancies. This discovery contradicted our understanding of the decreased invasive and metastatic abilities seen in mimic cells. Of course, focusing on one aspect at a time makes it hard to understand the overall picture. Some striking research has reported that xenogeneic rejection and cancer immunity might share a common molecular mechanism in the processes of induced necrosis and inflammation. Therefore, we further investigated this cross molecular mechanism in a model of immune rejection.

## 2 MATERIALS AND METHODS

### 2.1 Cell culture

1E8 cell lines were obtained from the Peking University Pathology Centre (Beijing, China) and were authenticated and checked for mycoplasma before experiments. Stable *nc886*‐overexpressing 1E8 cells or control cells were established as described previously and labeled with GFP. Cell fluorescence imaging and QRT‐PCR analyses were employed for verification.

### 2.2 Mice and immunohistochemistry

Our experimental aim was to investigate the mechanism by which tumor cells modulate immunity during blood metastasis. Hence, we built a blood metastatic model by injecting scramble or mimic 1E8 cells into the right atrium of mice (n = 12, 6 each group) after anesthesia. Animals were handled according to the guidelines and protocols supported by the Animal Care and Use Committees (supplementary animal ethnic).The important tissues of these mice were fixed with 4% paraformaldehyde for histological examination or immunohistochemistry. Hematoxylin‐eosin (H&E) staining was performed by a series of steps, including gradient dehydration, xylene clearing, wax embedding, dewaxing, and staining. For immunohistochemistry, tissue sections were routinely dewaxed, subjected to antigen retrieval and hydrogen peroxide incubation, blocked with goat serum for 0.5 hours, and incubated with the primary antibody overnight at 4°C. The primary antibodies were as follows: CD68 mouse monoclonal antibody (66231‐2‐Ig, Proteintech, 1:2000) and PSAT1 polyclonal antibody (A14124, ABclonal, 1:500). The secondary antibody (enzyme‐labeled goat anti‐mouse/rabbit IgG polymer, PV‐6000, ZSGB‐BIO) was incubated the next day for 0.5 hours. Finally, DAB substrate (ZLI‐9017, ZSGB‐BIO) was added to the sections for chromogenic analysis. The pathologic results were judged by an experienced pathologist.

### 2.3 Bone marrow (BM) isolates and macrophage counting

DMEM containing 10% fetal bovine serum was injected into the femur and tibia of C57BL/6 mice, and then BM cells were suspended and cultured at 37°C overnight. The next day, unattached cells were discarded by changing the media. Cell morphology was photographed under an inverted fluorescence microscope (T1‐SAM Nikon). Macrophage growth was evaluated by an EnoGeneCell^TM^ Counting Kit‐8 (CCK‐8) (E1CK‐000208, EnoGene).

### 2.4 Transmission electron microscope (TEM)

Spleen tissue (1 mm^3^ sections) were routinely anterior‐fixed in Karnovsky's solution for 24 hours at 4°C, washed three times for 10 minutes in 0.1 mol/L phosphate buffer (PB), posteriorly fixed in 1% osmium acid for 1.5 hours, and then washed three times with 0.1 MPB buffer for 10 minutes each time. Then, solutions of 50%, 70%, 80%, and 90% ethanol were used to dehydrate the tissue for 15 minutes each. Samples were embedded with pure resin embedding agent and polymerized as follows: 35°C for 16 hours, 45°C for 8 hours, 55°C for 14 hours, and 60°C for 48 hours. Electronic staining was performed with uranium acetate for 30 minutes, followed by washing with pure water three times, lead citrate staining for 2 minutes, NaOH washing once, and pure water washing twice. Thereafter, dehydration, embedding, solidification, slicing, staining, and filming were performed using a transmission electron microscope. Observations were made with a JEM‐1400 Plus electron microscope (Japan Electronics), and photographs were taken.

### 2.5 RNA extraction, QRT‐PCR and transcriptome sequencing

Total RNA extraction was performed according to the standardized process of the manufacturers’ instructions. We adopted standard kits, such as the TRIzol kit (Invitrogen), FastKing gDNA Dispelling RT SuperMix kit (KR118‐02 TIANGEN) and PowerUp^TM^ SYBR^TM^ Green Master Mix (A25742, Applied Biosystems). All QRT‐PCR primer sequences are found in Table S1.

The transcriptome experiments were managed by OE Biotech Co., Ltd. RNA sequencing was performed on three replicates. After RNA inspection using the Agilent 2100 Bioanalyzer (Agilent Technologies), the libraries were built using the TruSeq Stranded mRNA LT Sample Prep Kit (Illumina) and sequenced on the HiSeqTM 2500 sequencing platform. A total of 125 bp/150 bp paired‐end reads were generated. The Genome Browser build hg19 was used for read alignment. A *P* value < .05 and a fold change (FC) >2 or <0.5 were set as the thresholds for differentially expressed genes. GO enrichment and KEGG pathway analyses of DEGs were performed using R software based on the hypergeometric distribution. Datasets were deposited in the Gene Expression Omnibus under accession no. GSE143451.

### 2.6 Flow cytometry

Immune cells from animal tissue or blood were separated by using HISTOPAQUE^®^‐1077(10771, Sigma), and then the dissociated lymphocytes were resuspended in PBS and stained for surface markers for 20 minutes with antibodies: PerCP‐Cy^TM^ 5.5 rat anti‐mouse CD45 (550994, BD Biosciences, 1:200), FITC rat anti‐mouse CD8a (553030, BD Biosciences, 1:500), APC rat anti‐mouse CD11b (553312, BD Biosciences, 1:200), PE rat anti‐mouse F4/80 (565410, BD Biosciences, 1:200), APC anti‐mouse CD206 (MMR) (141707, BD Biosciences, 1:200), APC mouse anti‐human HLA‐ABC (555555, BD Biosciences, 1:200), APC mouse anti‐human HLA‐DR (560896, BD Biosciences, 1:125), and BV421 rat anti‐mouse CD119 (740032, BD Biosciences, 1:200). Flow cytometry data were obtained on an LSRFortessa, and data analysis was achieved through FlowJo software (Tree Star).

### 2.7 RNA immunoprecipitation (RIP) assay

All RIP experiments were carried out according to the instructions included with the RIP‐Assay Kit (No. RN1001, MBL). Anti‐MHC I (15240‐AP, Proteintech) or TAP1 (11114‐1‐AP, Proteintech) antibodies were used to pull down endogenous *nc886* complexes in mimic cells (1 × 10^8^ cells). Cells were fixed with 4 mL of 1% formaldehyde at 37°C for 10 minutes. After washing three times with cold PBS, the cell precipitate was lysed with lysis buffer containing 60 μL of 50× protease inhibitor and rotated at 4°C for 30 minutes. Short DNA fragments (100‐500 bp) were acquired using sonification under the following conditions: cycles: 99 times; continuous: 30 seconds; interval time: 30 seconds. Then, 100 μL of input was preserved. Four micrograms of antibody (anti‐TAP1, anti‐MHC I, or negative control IgG) and 30 μL of protein A/G magnetic beads were added to 250 μL of sonicated lysate and rotated at 4°C overnight. Finally, purified DNA/RNA was obtained by de‐crosslinking and digesting the proteins with proteinase K. QRT‐PCR was used to quantitatively evaluate the expression level of *nc886*.

### 2.8 Western blot analysis

After quantification of protein concentration using a BCA assay (Pierce), proteins were separated by SDS‐PAGE, transferred onto a PVDF membrane and then identified by immunoblot analysis with the appropriate primary antibodies at the following dilutions: anti‐MHCI (15240‐AP, Proteintech, 1:500) and anti‐TAP1 (11114‐1‐AP, Proteintech, 1:500). GAPDH (#4967, 1:8000) was used as an internal control. HRP + goat anti‐mouse IgG(H + L) (RS0001, ImmunoWay, 1:10 000) or anti‐mouse IgG (sc‐516102) was diluted to 1:8000. The membranes were developed using an enhanced chemiluminescence HRP substrate (WBKLS0500, Millipore Corporation Billerica) and then exposed to a MiniChemi 610 Imager (304002L, China).

### 2.9 Dual‐luciferase reporter assays

Cells were seeded in 24‐well plates, and plasmid transfection was performed the following day. Dual‐luciferase activity was analysed with a Dual‐Luciferase Reporter Assay System (E2920, Promega) in a microplate spectrophotometer (TriStar2 LB 942 Multimode Reader Berthold Technologies) according to the manufacturer's instructions.

### 2.10 Statistical analysis

All QRT‐PCR experiments were repeated at least three times to verify data accuracy. A two‐tailed Student's *t* test was used to analyse comparisons between two groups. The error index was calculated according to the standard deviation (SD). *P* < .05 was considered statistically significant.

## 3 RESULTS

### 3.1 Differentially downregulated genes between mimic and scramble cells were preferentially enriched in the immune system by transcriptome analysis

**FIGURE 1:**
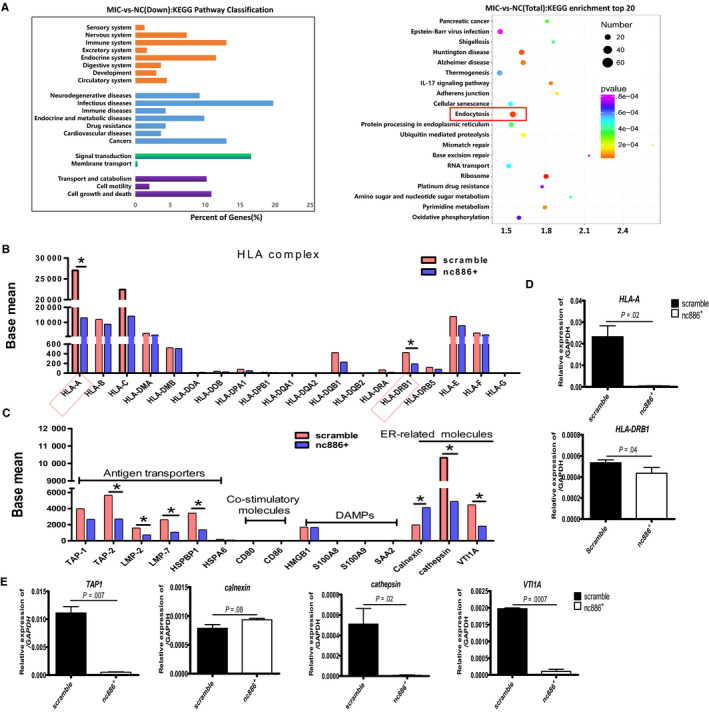
Transcriptome sequencing analysis of the differentially expressed genes between scramble and nc886^+^ cells. A, Left, KEGG pathway classification for differentially downregulated genes in nc886^+^ vs scramble cells, showing that the genes were mainly enriched in processes related to the immune system. Right, KEGG enrichment of the top 20 of the total DEGs, showing that the genes were mainly clustered in the endocytosis pathway. Differential transcripts from HLA molecules (B), antigen transporters, costimulatory molecules, DAMP‐related molecules, and endocytosis‐related molecules (C) were statistically analysed. D,E, Validation of DEGs by QRT‐PCR. Three independent experiments were performed

We checked differences of gene expression between the scramble and mimic cell lines by RNA sequencing analysis, and identified 2222 upregulated and 1546 downregulated differentially expressed genes (DEGs, *P*‐value adjustment < .05, and fold change > 2) in mimic vs scramble cells and performed an analysis of associated biological processes, cellular components, and molecular functions (Figure S1). An important mode of ncRNA regulation is reduced expression of mRNA targets; as such, we focused on the 1546 downregulated genes. Interestingly, these differentially downregulated genes were preferentially enriched in processes related to the immune system, infectious disease, cancer, and signal transduction by GO analysis (Figure 1A, left). Additionally, the DEGs were found to be associated with signaling pathways related to the endoplasmic reticulum network by KEGG analysis (Figure1A, right). We specifically analysed all the *HLA* molecules, antigen transporters, costimulatory factors, DAMPs, and molecules related to the endoplasmic reticulum network from the sequencing data and found that *nc886* significantly inhibited two HLA molecules (Figure 1B), four transporters, and two endoplasmic reticulum‐related molecules (Figure1C). Of the HLA molecules, only *HLA‐A* and *HLA‐DRB* had mRNA levels that were significantly downregulated (2.12‐fold and 2.28‐fold, respectively) (Figure 1B), and *TAP2, LMP‐2, LMP‐7,* and *HSP70*, but not *TAP1* and *HSP90,* were the transporter molecules that were significantly downregulated (2.12‐fold, 2.27‐fold, 2.50‐fold, and 2.56‐fold, respectively) in the nc886^+^ vs scramble groups (Figure 1C). *Calnexin*, *Cathepsin* and *VTI1A* (vesicle transport through interaction with t‐SNAREs 1A) were significantly upregulated (2.12‐fold) and downregulated (2.81‐fold 2.13‐fold) respectively. We validated these DEGs by QRT‐PCR. The expression patterns of most DEGs were consistent with the expression patterns suggested by RNA sequencing except for those of *TAP2*, *LMP‐2*, *LMP‐7*, *HSP70*, and *VTI1A* (Figure 1D,E). TAP1 was validated to be approximately 10‐fold downregulated in scramble cells vs mimic cells (Figure 1E). Presumably, *nc886* might disturb the antigen presentation of *HLA‐A or HLA‐DRB1* directly through transporter molecules in 1E8 cells.

### 3.2 Nc886 directly downregulates TAP1 and MHC‐I expression in tumor cell line

**FIGURE 2:**
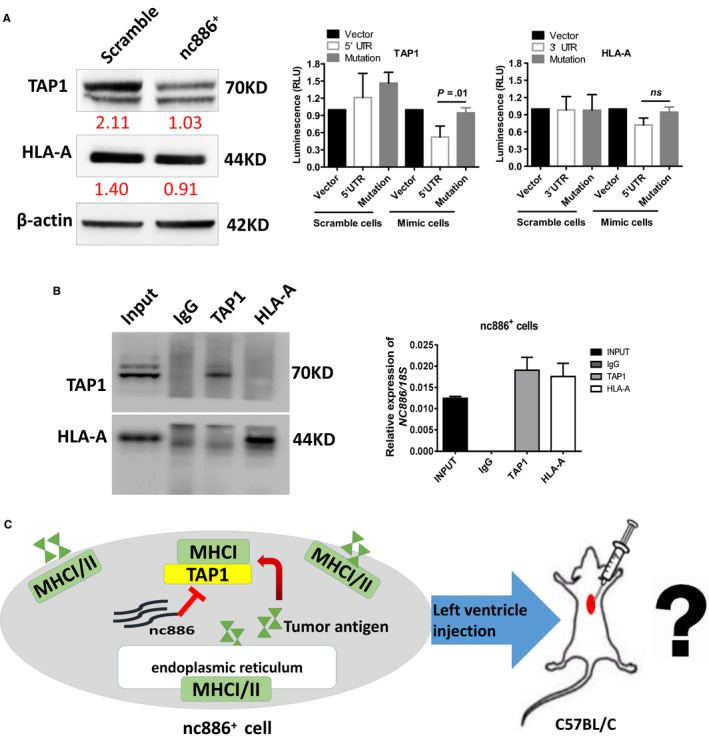
*Nc886* directly targets TAP1. A, Westernblot analysis of TAP1, HLA‐A and β‐actin in scramble and nc886^+^ cells. The gray scale ratio (normalized to β‐actin) was calculated by ImageJ software (left). TAP1 5’UTR and HLA‐A 3’UTR plasmids were constructed and transfected into scramble cells or nc886^+^ cells. Dual‐luciferase reporter assays were used to detect direct interactions with *nc886* (right). B, The RIP experiment validated the interaction between *nc886* and TAP1 or HLA‐A in nc886^+^ cell lines, and the left picture verified that the magnetic beads were successfully loaded with antibodies. C, Schematic diagram and problem of nc886 changing tumor antigen.

We detected that the UTR regions of TAP1 and HLA‐A were complementary sequences to the “seed” sequence of *nc886* through the Needleman‐Wunsch algorithm for global alignments, although the matches were imperfect (Figure S2). Next, we validated that the protein level of TAP1 was 2.1‐fold downregulated and the protein level of MHC‐1 was 1.56‐fold downregulated in scramble cells vs mimic cells (Figure 2A, left). We examined whether *nc886* directly downregulated TAP1 and MHC‐I expression by luciferase reporter and ChIP experiments. We created constructs containing exons 1 or 8 of TAP1 and HLA‐A recombinants and their mutants and transfected them into scramble cells without *nc886* expression or mimic cells with positive expression of *nc886*. The results showed that the luciferase value was reduced by approximately 4‐fold in cells that had been transduced with TAP1 vs mutant constructs in the mimic cell line after 24 hours of transfection; however, there was no significant difference in cells transduced with HLA‐A vs mutant constructs, and there was no change in scramble cell lines due to the lack of *nc886* (Figure 2A, right). Consistent with these results, we successfully amplified the *nc886* fragment in complex with TAP1 and MHC‐1 by CHIP experiments (Figure 2B), suggesting that they should be direct target molecules of *nc886*. The combined findings of the two experiments showed that *nc886* more directly downregulated TAP1 than HLA‐A. Apparently, nc886 can directly alter the processing and presentation of intrinsic antigens including tumor antigen in tumor cells, which raises the question of what kind of immune response occurs when such tumor cells are injected to immunocompetent mice (Figure 2C)?

### 3.3 Nc886 alleviates immune rejection from injection of tumor cells into the left ventricle of C57BL/C mice

**FIGURE 3:**
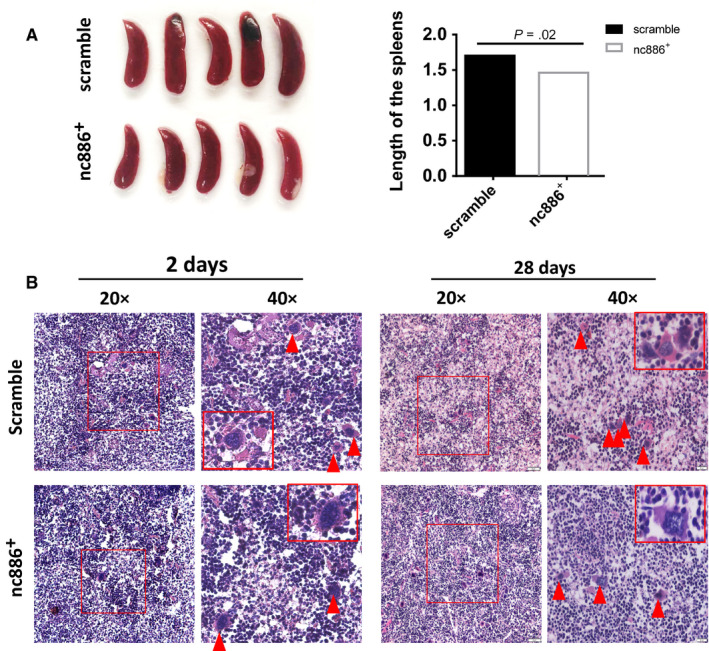
*Nc886* alleviates the splenic inflammation induced by xenogeneic rejection in C57BL/C mice. A, Left panel, spleens from the two groups are shown at 28 days (n = 5). Right panel, the lengths of the spleens were calculated in the two groups. B, Representative H&E staining images (20× and 40×) of mouse spleen tissues from the scramble and nc886^+^ groups at 2 and 28 days after injection of tumor cells into the left ventricle. The rectangles and triangles indicate the macrophages in the highlighted region. Scale bar, 50 μm and 20 μm

Using immunodeficient mice as an animal model limited our experiments to analyse the role of *nc886* in immune function. Thus, in vivo, 1 × 10^5^ cancer cells were injected into the left ventricle of C57BL/6 mice to investigate the differences between mimic and scramble cells in terms of immune rejection responses after 2 and 28 days. Histological analysis detected less immune rejection after mimic cell injection than after scramble cell injection. Inflammatory edema of the spleen, liver, lung, and bone occurred in the scramble group, especially splenomegaly, which lasted for 90 days; however, the nc886^+^ group saw only slight inflammatory edema (Figure 3B; Figure S3). H&E staining showed that the spleens from the control groups were obviously necrotic, and splenic knots were lost compared with those from the nc886^+^ groups. A large number of macrophages were recruited to necrotic or inflammatory regions in both groups. The nc886^+^ group maintained intact splenic nodules (Figure 3A). The results suggested that compared with mimic cells, scramble cells caused a relatively intense organ inflammatory response associated with rejection, especially in the spleen (Figure 3). Clearly, *nc886* might decrease the immune rejection response which is closely related to downregulation of MHCI expression in the nc886^+^cells.

### 3.4 Macrophages recruited to the spleen exhibit positive expression of both CD68 and prostate‐specific antigen(PSA)

**FIGURE 4:**
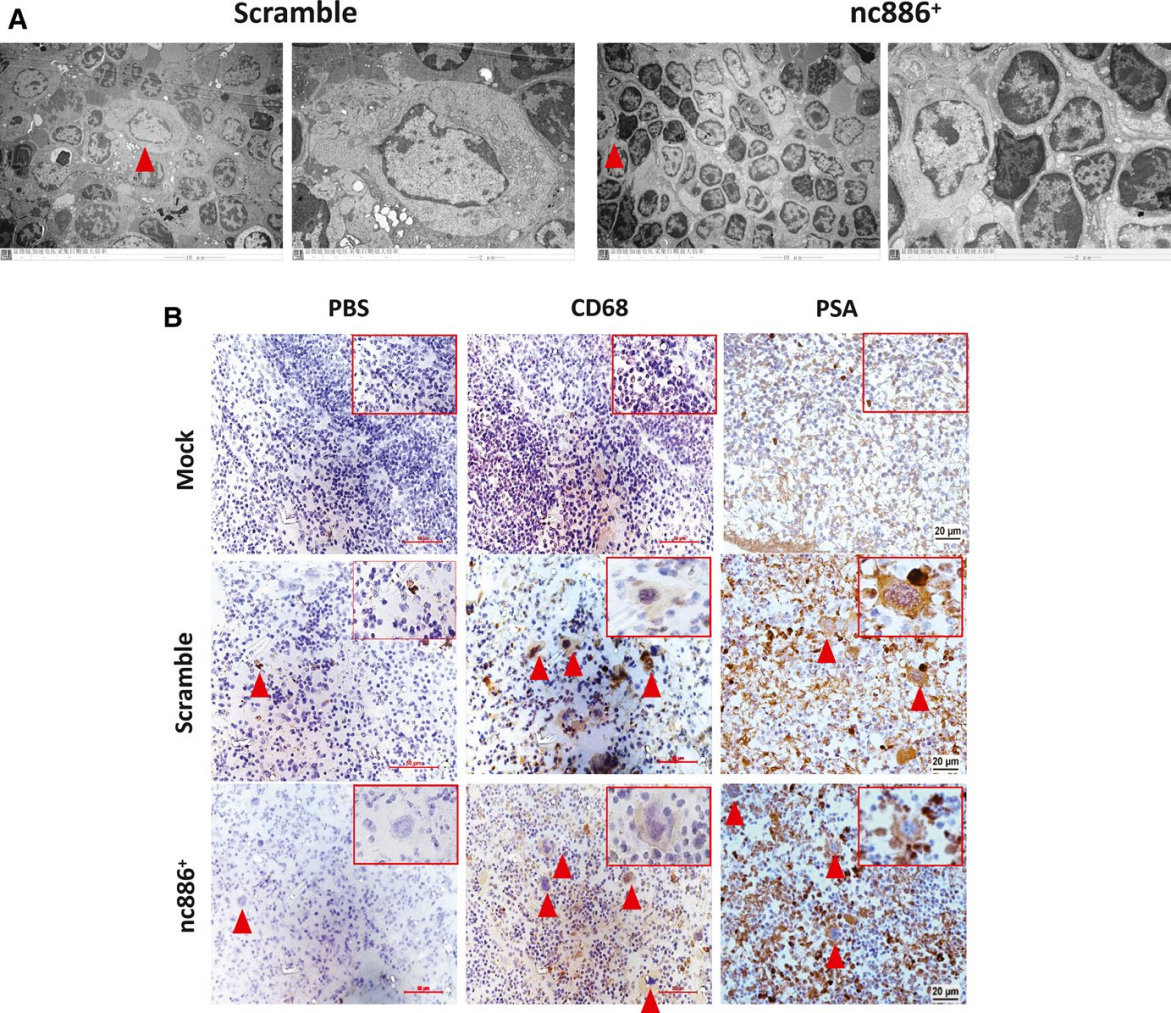
Macrophages with engulfed tumor cells accumulate in inflammatory regions of the spleen. A, Transmission electron microscope images of representative macrophages from spleen tissues (n = 3). Scale bar, 10 μm and 2 μm. Triangles indicate enlarged macrophages. B, Representative immunohistochemical images for CD68 and PSA staining in mouse spleen samples (40×) (n = 6). The triangles highlight macrophages in inflammatory regions. The rectangles indicate typical macrophages with PSA or CD68 staining. PBS was used as a negative control. Scale bar, 20 μm. Mouse samples at 28 days are shown in this panel

The recruitment of innate immune cells to the spleen has been known to be important in the early‐stage rejection reaction,^21^, ^22^ so we focused on observing the micromorphological changes and characteristics of macrophages that were recruited to the splenic inflammatory region by transmission electron microscopy (TEM) and immunohistochemistry. Interestingly, the ultrastructures of macrophages in the scramble group and nc886^+^ group were somewhat different from those in normal mice, for example, mitochondrial depolarization, swollen and extremely electron‐dense mitochondria, and nucleolus loss in the scramble group (Figure 4A). CD68 is the most reliable marker of macrophages, and PSA is a specific marker of prostate cancer. Immunohistochemistry analysis of macrophages in the two groups indicated strong CD68 and PSA staining that persisted in areas of splenic inflammation for 28 days after 1E8 cell injection (Figure 4B). This result suggested that macrophages participated in immune rejection in the spleen.

### 3.5 Nc886 might activate T cells or macrophages to destroy the tumor cells

**FIGURE 5:**
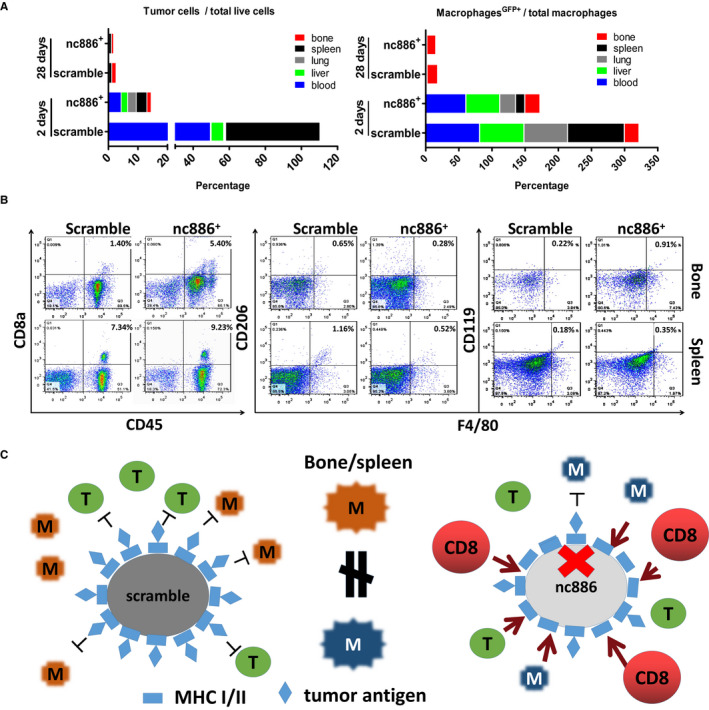
Nc886 promoted CD45^+^CD8a^+^ and CD119^+^F4/80^+^ cells increasing in bone marrow. A, The percentage of tumor cells with the GFP tag in the total surviving cells distributed in the bone, spleen, lung, liver, and blood (left) and the percentage of GFP^+^ tissue macrophages in total macrophages in the bone, spleen, lung, liver, and blood (right). B, Flow cytometry revealed different clusters of lymphocytes, such as CD45^+^CD8a^+^, CD206^+^F4/80^+^, and CD119^+^F4/80^+^ cells, isolated from the bone and spleen of mice at 2 and 28 days (n = 6). C, The mechanical diagram. nc886 can activate CD8a T cells to destroy tumor cells through this mechanism of restricting tumor antigen loading. Activated macrophages from bone marrow and spleen tissue between scramble groups and nc886^+^ group has some differences in micromorphology and characteristics

Furthermore, we investigated the distribution of tumor cells labeled with a GFP tag after left ventricle injection and the distribution of CD45^+^F4/80^+^GFP^+^ macrophages through flow cytometric screening. GFP^+^ cells in the nc886^+^ group were significantly decreased compared with those in the scramble group (Figure 5A, left). At 2 and 28 days, the proportion of GFP^+^ cells was highest in the spleen (52.7%), followed by the blood (49.7%), liver (7.4%), lung (0.7%),and bone (0.71%), in the scramble group and was highest in the blood (4.2%), followed by the spleen (3.6%), lung (2.9%), liver (2.2%),and bone (1.43%), in the nc886^+^ group after 2 days. However, at 28 days, the highest proportions of GFP^+^ cells were in the bone (0.99% and 0.91%, respectively) and spleen (1.46% and 0.71%, respectively) in the scramble and nc886^+^ groups (Figure 5A, left). The highest proportion of CD45^+^F4/80^+^GFP^+^ macrophages in total macrophages (CD45^+^F4/80^+^) was found in the spleen (85.6%), followed by liver (66.9%), the blood (65.7%), lung (65.7%),and bone (22.2%), in the scramble group and was found in the blood (60.6%), followed by the liver (51%), lung (23.8%), bone (22.9%), and spleen (14.1%), in the nc886^+^ group at 2 days. After 28 days, CD45^+^F4/80^+^GFP^+^ macrophages were also found in the bone (16.1% and 13.5%, respectively) and spleen (1.94% and 1.71%, respectively) in the scramble and mimic groups (Figure 5A, right). These data demonstrated a differential distribution of GFP^+^ cells and CD45^+^F4/80^+^GFP^+^ macrophages in the two groups at 2 or 28 days (Figure 5A). Furthermore, we identified aCD8a^high^ population in the nc886^+^ group that was not present in the scramble group in bone marrow aspirates but not in splenic homogenates; in contrast, CD206^high^ cells were found in both the bone marrow and spleen in the scramble group but not in the nc886^+^ group. Additionally, aCD119^high^ (IFN‐γ receptor) population was identified in the bone marrow and spleen in the nc886^+^ group (Figure 5B). iNOS was not detected in either of the two groups (data not shown). The diagram explained that nc886 can promote the production of killer T cells (CD8a) due to its restriction for tumor antigen presentation. In addition, activated macrophages from bone marrow and spleen tissue between sramble and nc886^+^ groups also exists differences in micromorphology and characteristics (Figure 5C).

### 3.6 Nc886 decreases the M2‐like macrophage population in vivo

**FIGURE 6:**
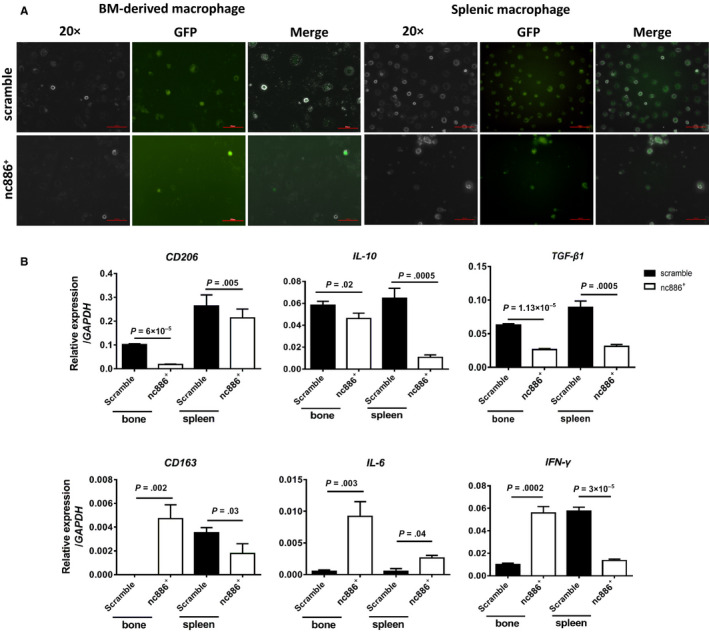
*Nc886* decreases M2‐like macrophages. A, Representative fluorescence microscopy images of BM‐derived and splenic macrophages (macrophage‐SP) and macrophages with a GFP tag and merge picture, indicating that more GFP^+^ macrophages were present in the bone and spleen of the scramble group than in the respective locations in the nc886^+^ group. B, QRT‐PCR was employed to examine the mRNA expression of *CD206*, *IL‐6*, *IL‐10*, and *IFN‐γ* in BM‐derived macrophages and splenic macrophages from the two groups of 28‐day‐old mice (n = 6). Data are shown as the mean ± SD

We noticed that there were many more GFP^+^ BM‐derived and splenic macrophages in the scramble group than in the nc886^+^ group under a fluorescence microscope (Figure 6A). In combination with the TEM observations (Figure 4A), which showed that these macrophages in the spleen tissues of the scramble group had larger sizes and more abundant lysosomes and phagocytes than those in the nc886^+^ group, these findings suggest that these macrophages that engulfed tumor cells should be one of the causes of chronic inflammation. Using QRT‐PCR, we found higher expression of *CD163*, *IL‐6*, and *IFN‐Ƴ* in the bone marrow of the nc886^+^ group than in the bone marrow of the scramble group; we also found lower expression of *CD163* and *IFN‐Ƴ*, but not *IL‐6*, in spleen tissue homogenates from the nc886^+^ group than the respective levels in spleen tissue homogenates from the scramble group (Figure 6B, lower). The expression of *CD206, IL‐10*, and *TGF‐β1* in the nc886^+^ group was significantly lower than that in the scramble group in both spleen tissue homogenates and bone marrow samples (Figure 6B, upper). These findings on M1 and M2 macrophages suggest that *nc886* reduces the M2 macrophage population. What factors promote M2‐like macrophage polarization in the scramble group?

### 3.7 TGF‐β1 drives the process of M2 polarization in bone marrow and spleen

**FIGURE 7:**
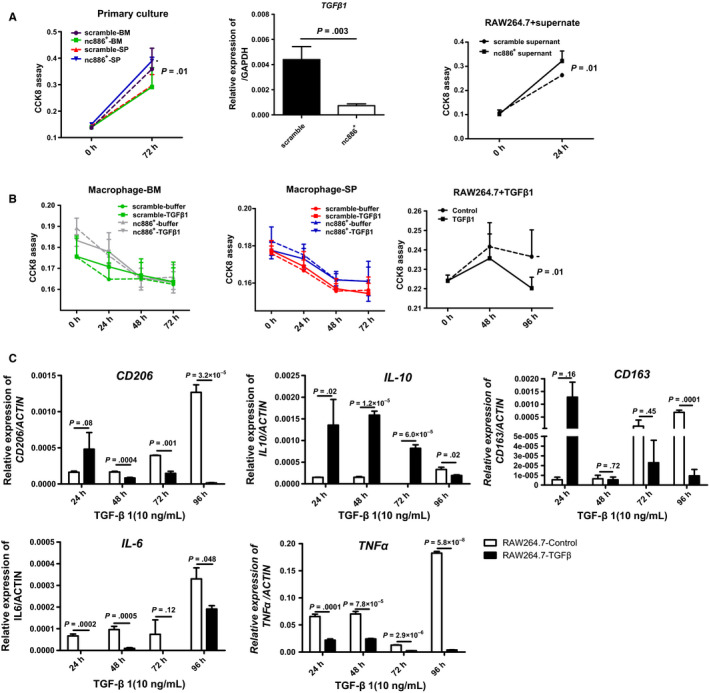
TGF‐β1 is vital in the process of M2 polarization. A, CCK8 assays were adopted to examine the growth of BM‐derived and splenic (SP) macrophages (left), and of RAW264.7 cells cultured in supernatant from scramble cells or nc886^+^ cells respectively (right). Scramble cells overexpressed TGF‐β1 fivefold more than mimic cells by QRT‐PCR (middle). B, The growth of primary macrophages or RAW264.7 cells upon TGF‐β1 (10 ng/mL) stimulation. C, TGF‐β1 affected the mRNA levels of *CD206, CD163*, *IL‐6*, *IL‐10*, and *TNF‐α* in RAW264.7 cells. At least three independent experiments are shown as the mean ± SD

**FIGURE 8:**
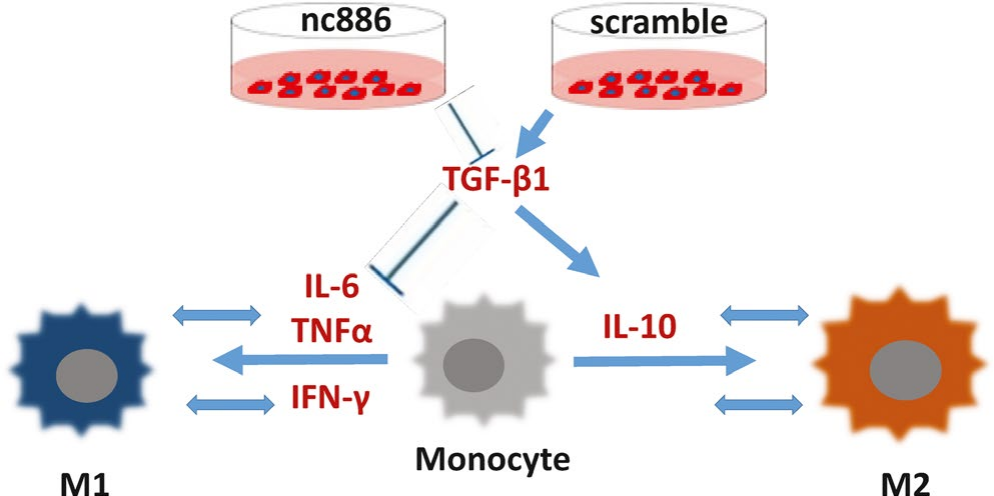
The mechanical diagram in the process of M1/M2 polarization. Our data support an important regulatory role of TGF‐β1 in macrophage polarization. Scramble cells can secrete a high concentration of TGF‐β1 to induce M2‐like phenotype in inflammation region. TGF‐β1 increases the expression of *IL‐10* and decreases the expression of *IL‐6* and *TNF‐α*, which are secreted by monocytes or macrophages. In contrast, nc886 can activate M1‐like phenotype through the mechanism of TGF‐β1 inhibition

The growth of the splenic macrophages, not BM macrophages, in the two groups was significantly different, with the splenic macrophages in the nc886^+^ group proliferating faster than those in the scramble group (Figure 7A, left). We also found that scramble cells expressed higher levels of TGF‐β1 than mimic cells (Figure 7A, middle). RAW264.7 grew more slowly cultured in the supernatant of scramble cells than of nc886^+^ cells (Figure 7A, right). Additionally, in vitro, 10 ng/mLTGF‐β1 inhibited the growth of primary macrophages, either spleen‐ or BM‐derived, in the two groups (Figure 7B), and inhibited the growth of the macrophage cell line RAW264.7 (Figure 7B, right). Considering the higher mRNA level of TGF‐β1 in the BM samples and splenic homogenates of the scramble group (Figure 6B), we concluded that TGF‐β1 slowed the growth of macrophages in the scramble group. Meanwhile, we hypothesize that TGF‐β1 also induced an M2/M1 transition in our model. Indeed, after treatment with 10 ng/mL TGF‐β1, RAW264.7 cells rapidly began secreting *IL‐10*, which lasted for 72 hours; however, *CD206* or *CD163*mRNA levels were increased shortly after 24 hours and then gradually decreased (Figure 7C, upper). Conversely, 10 ng/mLTGF‐β1 continuously decreased *IL‐6* and *TNF‐α* mRNA levels (Figure 7C, lower). These data suggest the vital role of TGF‐β1 in the process of M2 polarization. The mechanical diagram is listed in Figure 8. It explains that nc886 reduces the M2 polarization of macrophages by reducing the expression of TGF‐β1. TGF‐β1 upsets the balance of M1/M2 transition by inhibiting the IL‐6 and TNF‐α secretion and promoting IL‐10 release.

## 4 DISCUSSION

Notably, our transcriptome sequencing data showed that nc886 downregulated the expression of *HLA‐A* and *HLA‐DRB* related to the endocytosis pathway. We subsequently tested the changes in some endoplasmic molecules associated with antigen processing. In accordance with our expectations, *nc886* reduced the expression of *TAP1*, *cathepsin* (early endoplasmic molecule), and *VTI1A* (late endoplasmic molecule) and increased *calnexin* mRNA levels.^23^, ^24^ We additionally noticed that *nc886* could directly downregulate the expression level of TAP1, as validated by luciferase reporter and ChIP assays. Hence, it is postulated that *nc886* alters antigen processing and presentation by decreasing the expression of some HLA molecules and antigen transporters.

MHC I/II downregulation by *nc886* should be one of reasons to alleviate tumor cellular immunological rejection in immunocompetent mouse models. Considering that the homology of the HLA‐A, HLA‐B, and DRB1 genes between humans and mice is 64.36%, 68.61%, and 76.63%, respectively, the MHC mismatch‐mediated host T cell response should not be neglected. In addition, the innate immune system is the fundamental protective barrier of our body against exogenous intrusion, especially macrophages, which are vital cells for xenogeneic rejection.^25^ Our model showed a significant increase in macrophages and abnormal macrophage polarization in the spleen tissues of the scramble group.

We tracked the tissue distribution of bone‐metastatic 1E8^GFP+^ cells entering host blood through flow cytometric analysis at 2 and 28 days. In both the scramble group and nc886^+^ group, 1E8^GFP+^ cells were mainly distributed in the blood and spleen at 2 days and in the bone and spleen at 28 days. However, the number of total 1E8^GFP+^ cells in the nc886^+^ group was dramatically less than that in the scramble group at 2 or 28 days, suggesting that more mimic cells than scramble cells were removed by immune‐mediated destruction in the early stage. Further findings showed that the number of CD8a^+^ T cells in the bone, but not in the spleen, were dramatically increased by 3.86‐fold in the nc886^+^ group compared with that in the scramble group. These data support the idea that many mimic cells were attacked early by CD8^+^ T cells in the blood. Additionally, splenic rejection in the scramble group was more serious than that in the nc886^+^ group, lasting 90 days. Notably, splenic macrophages showed morphological changes by TEM between the two groups. The flow cytometry and QRT‐PCR results suggest that many more M2 macrophages (pro‐regenerative phenotype) were distributed in the bone and spleen of the scramble group than the number in the nc886^+^ group; however, many more M1 macrophages (pro‐inflammatory) were found in the nc886^+^ group than in the scramble group. Macrophage polarization states often depend on a specific set of cytokines in the tissue microenvironment, including tumor necrosis factor (TNF) and TGF‐β1.^26^, ^27^ TGF‐β1 is a well‐documented factor that polarizes naïve macrophages to the M2 phenotype.^28^ In vitro, although TGF‐β1 inhibited the growth of primary macrophages and RAW247.1 cells, it promoted *CD206*, *CD163*, and *IL‐10* upregulation in RAW247.1 cells and inhibited *IL‐6* and *TNF‐α* expression. Surely, TGF‐β1 is a vital factor in maintaining the M2‐like phenotype of the scramble group. Interestingly, we did not find tumor colonies in important organs, such as the bone, lung, spleen, and liver, even after 120 days, so we believe that most tumor cells are ultimately eliminated by the host's immune system. However, GFP^+^ or PSA‐positive M2 macrophages still existed in the splenic inflammation region. This is an important reason for chronic inflammation in the spleen of the scramble group. These findings help us clarify why splenic inflammation in the scramble group is more serious than that in the nc886^+^ group.

However, why mimic cells with MHC I/II downregulation can still activate T cells and macrophages but scramble cells with MHC I overexpression cannot remain unknown. We postulate that some specific tumor antigens help them escape immune surveillance. Tumor cells, as a special type of APC, often process self‐antigen peptides through the proteasome and endoplasmic reticulum system.^29^ Tumor antigens, such as differentiation antigens, mutational antigens, overexpressed cellular antigens, and viral antigens, contribute to immunological tolerance.^30^ However, our previous study showed that PSA levels did not differ between scramble cells and mimic cells. We found that the levels of *CD274 (PDL1)*, *CD80*, *CD86*, *CD47*, and *DAMP* molecules in scramble cells were not higher than those in mimic cells. Noteworthily, in our study model, scramble cells overexpressed TGF‐β1 fivefold more than mimic cells, and high levels of TGF‐β1 were also found in bone marrow aspirates and spleen homogenates from the scramble group. Accumulating evidence confirms that TGF‐β1 is an important cause of immunosuppressive effectors in the scramble group. Certainly, some new tumor antigens need to be recognized in the future.

Many studies have reported that immunological tolerance is associated not only with tumor antigen mutations but also with low expression of MHC or TAP molecules.^31^, ^32^, ^33^, ^34^ Nevertheless, with the notable exception of MHC I downregulation, the opposite effect was observed in cancer in our model. We believe that *nc886* affects tumor antigen loading and presentation on the surface of tumor cells by directly decreasing the protein levels of HLA‐A and TAP1 transporters. TAPs mainly transport antigens from the cytoplasm into the endoplasmic reticulum for processing. We suggest a new point of view in which *nc886* decreases the invasive ability of prostate cancer cells by altering tumor antigen processing and presentation.

## CONFLICT OF INTEREST

None declared.

## AUTHOR CONTRIBUTIONS

Hui Ma, Ying Zhou, Jia‐jie Yang, and Rong‐Hui Yang performed the experiments, Miao Wang, Li‐Yong Wang and Min‐jie Wen analyzed the data, Lu Kong designed the experiments and prepared the manuscript and submitted the final manuscript.

## Supporting information

Fig S1

Fig S2

Fig S3

Table S1

Supplementary Material

## Data Availability

The data used to support the findings of this study are available from the corresponding author upon request.
