# Supplementary figures and images for: Noncoding RNA 886 alleviates tumor cellular immunological rejection in host C57BL/C mice
Source: Cancer Med. 2020 May 31;9(14):5258–71. doi: 10.1002/cam4.3148 (PMC7367629; doi:10.1002/cam4.3148)

# A

## Statistic of Differently Expressed gene

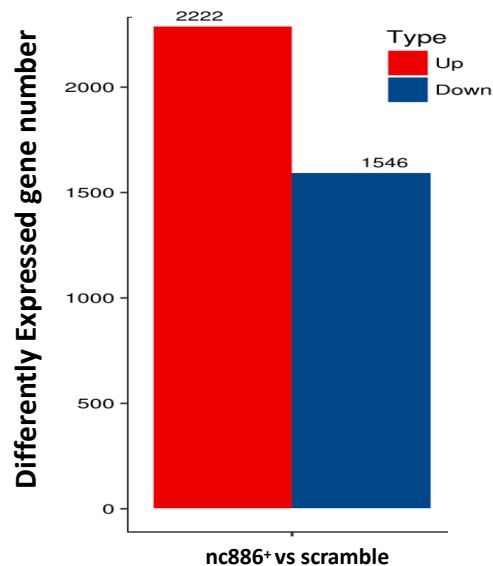

# B

## Gene Ontology nc886+ vs scramble

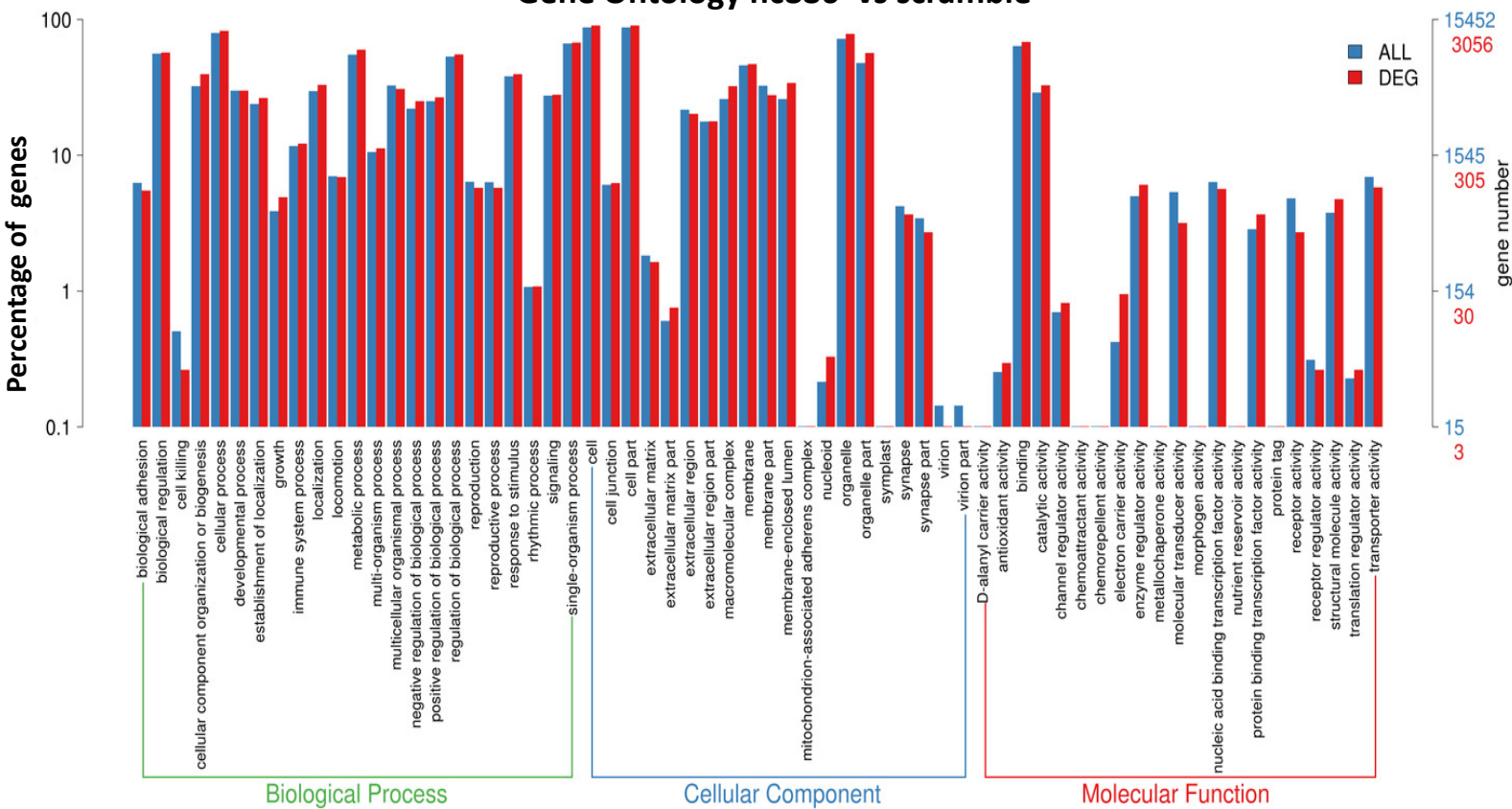

Supplement: Supplementary file 1 — Fig S1 [file CAM4-9-5258-s001.pdf]

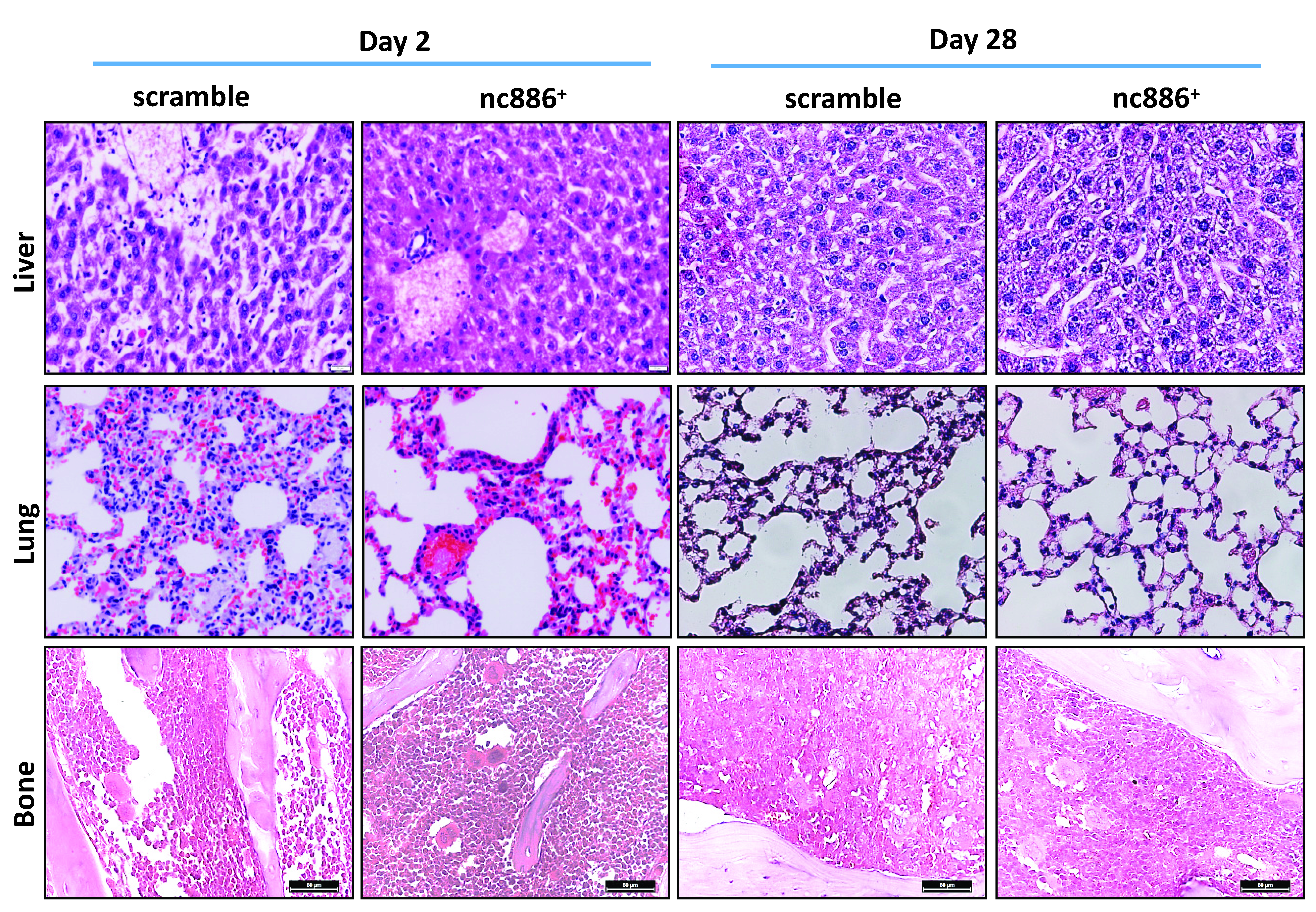

Supplement: Supplementary file 3 — Fig S3 [file CAM4-9-5258-s003.tiff]
