# Supplementary material for: Noncoding RNA 886 alleviates tumor cellular immunological rejection in host C57BL/C mice
Source: Cancer Med. 2020 May 31;9(14):5258–71. doi: 10.1002/cam4.3148 (PMC7367629; doi:10.1002/cam4.3148)
Supplement: Supplementary file 2 — Fig S2 [file CAM4-9-5258-s002.pdf]

A

(Query, HLA-A, transcript variant 1, mRNA,1261-1440, exon 8)

```
Query  1261  ACCACCCCACCCCCATGTCCACCATGACCCTCTTCCCACGCTGACCTGTGCTCCCTCCCC  1320
             |||                                   ||| |    |||||
Sbjct  77    ACC-----------------------------------CGCGG----GTGCTT-------  90

Query  1321  AATCATCTTTCCTGTTCCAGAGAGGTGGGGCTGAGGTGTCTCCATCTCTGTCTCAACTTC  1380

Sbjct        ------------------------------------------------------------

Query  1381  ATGGTGCACTGAGCTGTAACTTCTTCCTTCCCTATTAAAATTAGAACCTTAGTATAAATT  1440
                    |||||                 |||| |||
Sbjct  91    -------ACTGA-----------------CCCTTTTA-----------------------  103
```

B

(Query, TAP1, transcript variant 1, mRNA,241-480, exon 1)

```
Query  241   GTAGGGGAGGACTCGGCGGTACCCGGAGCTTCAGGCCCCACCGGGGCGCGGAGAGTCCCA  300
                                                      |||| ||    ||||
Sbjct  1                                              CGGGTCG----GAGT----  11

Query  301   GGCCCGGCCGGGACCGGGACGGCGTCCGAGTGCCAATGGCTAGCTCTAGGTGTCCCGCTC  360
                                                     |||||| ||
Sbjct  12    ----------------------------------------TAGCTCAAG-----------  20

Query  361   CCCGCGGGTGCCGCTGCCTCCCCGGAGCTTCTCTCGCATGGCTGGGGACAGTACTGCTAC  420
                 ||| | ||
Sbjct  21    ----CGGTTACC------------------------------------------------  28

Query  421   TTCTCGCCGACTGGGTGCTGCTCCGGACCGCGCTGCCCCGCATATTCTCCCTGCTGGTGC  480
```

Query ID NM_002116.8 (A); NM_000593.5 (B)

Subject ID NR_030583.3 , Homo sapiens vault RNA 2-1 (VTRNA2-1)
