# Supplementary material for: Noncoding RNA 886 alleviates tumor cellular immunological rejection in host C57BL/C mice
Source: Cancer Med. 2020 May 31;9(14):5258–71. doi: 10.1002/cam4.3148 (PMC7367629; doi:10.1002/cam4.3148)
Supplement: Supplementary file 4 — Table S1 [file CAM4-9-5258-s004.rtf]

Table S1 QRT-PCR, reporter gene and chip primer sequences used in this study

gene accession	gene name	primers	5'-3'	
NR_030583.3 	nc886	Forward primer	AAGGGTCAGTAAGCACCCGCG	
		Reverse primer	CGGGTCGGAGTTAGCTCAAGCGG	
NR_003286.4	18s	Forward primer	GGACAGGATTGACAGATTGAT	
		Reverse primer	AGTCTCGTTCGTTATCGGAAT	
NM_013693.3	TNFα mouse	Forward primer	GATCGGTCCCCAAAGGGATG	
		Reverse primer	GTTTGCTACGACGTGGGCT	
NM_010548.2	IL10 mouse	Forward primer	TAACTGCACCCACTTCCCAG	
		Reverse primer	AGGCTTGGCAACCCAAGTAA	
NM_001314054.1 	IL6 mouse	Forward primer	CAACGATGATGCACTTGCAGA	
		Reverse primer	TGTGACTCCAGCTTATCTCTTGG	
NM_008625.2 	CD206 mouse	Forward primer	GTGGAGTGATGGAACCCCAG	
		Reverse primer	CTGTCCGCCCAGTATCCATC	
NM_001170395.1	CD163 mouse	Forward primer	CTGCTGTCACTAACGCTCCT	
		Reverse primer	TCATTCATGCTCCAGCCGTT	
NM_011577.2	TGFβ1 mouse	Forward primer	AGCTGCGCTTGCAGAGATTA	
		Reverse primer	AGCCCTGTATTCCGTCTCCT	
NM_010511.3	IFN mouse	Forward primer	GTAGCCTCACCGCCTATCAC	
		Reverse primer	GGGCCTCTCCTGTGAGTCTA	
NM_007393.5	actin mouse	Forward primer	CACTGTCGAGTCGCGTCCA	
		Reverse primer	GACCCATTCCCACCATCACA	
NM_001242758.1	MHC I human	Forward primer	TTCTTCACATCCGTGTCCCG	
		Reverse primer	CTCGGTCAGTCTGTGAGTGG	
NM_019111.5	MHC II human	Forward primer	GTTTACGACTGCAGGGTGGA	
		Reverse primer	GCTTTTGCGCAATCCCTTGA	
NM_000660.6	TGFβ1 human	Forward primer	GGGCACTGTTGAAGTGCCTTA	
		Reverse primer	GCAGGAACTCCTCCCTTAACC	
NM_001290043.2 	TAP2 human	Forward primer	CTAGCTAGCTCAGCGCTGAAGCAG	
		Reverse primer	ATAAGAATGCGGCCGCAAATATCCATTGAAAT	
NM_000593.5	TAP1 human	Forward primer	GCTGTAAGCAGTGGGAACCT	
		Reverse primer	GTGCGGTCCAGGTACTCAAA	
NM_004079.5	Cathepsin human	Forward primer	TTGTGTGCTCTTGGTGTGCT	
		Reverse primer	TCTTCACTGGTCATGTCTCCC	
NM_001024649.1	Calnexin human	Forward primer	GCCTCCGCCTCTCTCTTTAC	
		Reverse primer	TGGGAGATGAAGGAGGAGCA	
NM_001318205.1	VTI1A human	Forward primer	AGAGAACCAGAGGGCACATC	
		Reverse primer	TCTTGCTCTTACCTTCGCAAC	
NM_001289726.1	GAPDH human	Forward primer	GGGTCCCAGCTTAGGTTCAT	
		Reverse primer	TACGGCCAAATCCGTTCACA	
NM_000593.5	TAP1 normal human	Forward primer	AATTATGCTAGCCGCTTTCCCCTAAAT	
		Reverse primer	TGATACTCTAGACTAGAGCTAGCCATT	
NM_000593.5	TAP1 mutation human	Forward primer	AATTATGCTAGCCGCTTTCCCCTAAAT	
		Reverse primer	TGATACTCTAGATAGCCATTGGCACTC	
