# Supplementary material for: Noncoding RNA 886 alleviates tumor cellular immunological rejection in host C57BL/C mice
Source: Cancer Med. 2020 May 31;9(14):5258–71. doi: 10.1002/cam4.3148 (PMC7367629; doi:10.1002/cam4.3148)
Supplement: Supplementary file 5 — Supplementary Material [file CAM4-9-5258-s005.rtf]

Supplementary figure legends
Figure S1 Numbers and Gene Ontology analysis of DEGs in nc886+ vs. scramble cells
(A) DEGs were identified using the estimateSizeFactors and nbinomTest of the DESeq R package (v1.24.0). p value < 0.05 and FC >2 or FC < 0.5 were set as the thresholds for significant differential expression. (B) GO analysis of All gene and DEGs was performed using R based on the hypergeometric distribution.

Figure S2 Schematic diagram of seed sequences matched through the Needleman-Wunsch algorithm for global alignments.
Seed sequences of the nc886 (NCBI Reference Sequence: NR_030583.3) binding sites within the human HLA-A (A, NM_002116.8) and TAP1 (B, NM_000593.5) mRNAs, with substitutions in nc886 binding sites marked in red rectangles.

Figure S3 Nc886 alleviates the inflammation of the liver, lung and bone in C57BL/C mice induced by xenogeneic rejection
Representative H&E staining images (20x) of mouse lungs, livers and bones from the scramble and nc886+ groups at 2 and 28 days after injection of tumour cells into the left ventricle. Scale bar, 50 μm.
